# Topographical Organization of Prefrontal Cortex and Adjacent Areas Projections to the Dorsomedial Caudate–Putamen in Rats: A Retrograde Tracing Study

**DOI:** 10.3390/brainsci15040398

**Published:** 2025-04-15

**Authors:** Christopher L. Robison, Theodore Kazan, Rikki L. A. Miller, Tyler Allen, Jason S. Hensley, Sergios Charntikov

**Affiliations:** Department of Psychology, University of New Hampshire, Durham, NH 03824, USA

**Keywords:** prefrontal cortex, dorsomedial caudate–putamen, retrograde tracing, Fluoro-Gold, neural connectivity, topographical organization

## Abstract

The dorsomedial caudate–putamen (dmCPu), a key input structure of the basal ganglia, plays a crucial role in goal-directed behaviors and the transition to habits. The functional specialization of the dmCPu along its anteroposterior axis suggests that distinct prefrontal cortex (PFC) subregions may differentially contribute to these processes. However, the precise topographical organization of PFC and adjacent areas projections to the anterior and posterior dmCPu remains poorly understood. We employed retrograde tracing using Fluoro-Gold to map the projections from PFC subregions and adjacent areas to the anterior and posterior dmCPu in male Sprague Dawley rats. Histological verification and immunohistochemical labeling were conducted to confirm injection sites and neuronal labeling. Quantitative analyses were performed to assess the effects of injection site placement (anterior vs. posterior dmCPu), laterality (ipsilateral vs. contralateral), and cortical subregion on projection density. The posterior dmCPu received significantly higher projection densities than the anterior dmCPu, with a pronounced ipsilateral dominance across all cortical subregions. Among the subregions examined, the cingulate cortex exhibited the highest number of labeled neurons projecting to the dmCPu, with distinct patterns of connectivity between anterior and posterior injection sites. Notably, motor and somatosensory cortical projections were more prominent in the posterior dmCPu, whereas cingulate projections demonstrated robust anteroposterior and lateralized differences. These findings provide a comprehensive map of the topographical organization of cortical inputs to the dmCPu, highlighting differential connectivity patterns that may underlie distinct functional roles in goal-directed and habitual behaviors. This work advances our understanding of corticostriatal circuits and their relevance to adaptive behaviors and neuropsychiatric disorders.

## 1. Introduction

The prefrontal cortex (PFC) is integral to orchestrating a wide range of cognitive and behavioral functions, including working memory, attention regulation, problem-solving, and the coordination of motor responses [1]. These higher-order processes rely on intricate neural networks, with the PFC serving as a hub for interactions with subcortical structures such as the dorsomedial caudate–putamen (dmCPu) [2,3]. Early studies on PFC efferent pathways demonstrated the topographical organization of corticostriatal projections, establishing the foundational framework for investigating PFC-striatal connectivity [3,4]. The dmCPu, as a principal input structure of the basal ganglia, plays a pivotal role in goal-directed behaviors and the transition to habitual actions [5,6,7]. Notably, research has revealed that the functions of the dmCPu vary along its anteroposterior axis, with distinct cortical regions projecting to specific subregions of the dmCPu to regulate these behaviors [7,8,9]. Despite the importance of these corticostriatal connections, the precise topographical organization of inputs from different PFC subregions to the anterior and posterior dmCPu remains poorly understood. A better understanding of corticostriatal organization is critical for unraveling the neural circuitry underlying decision-making, cognitive flexibility, and habit formation.

Emerging evidence suggests that specific prefrontal subregions contribute uniquely to behavioral regulation through their connections with the dmCPu [8,10,11,12]. For example, the prelimbic cortex (PL) has been implicated in goal-directed actions, whereas the infralimbic cortex (IL) supports the formation and execution of habitual responses [11,13,14]. These differential roles are thought to stem from distinct anatomical and functional connectivity patterns between the PFC and the anterior and posterior dmCPu. Nevertheless, the precise mechanisms mediating these interactions are not well understood, demonstrating the need for detailed studies to map these projection patterns and their behavioral implications. By delineating these pathways, researchers can better target those pathways, and thus gain deeper insights into how corticostriatal circuitry governs adaptive behaviors and how its dysfunction contributes to neuropsychiatric disorders.

Early tracing studies inferred synaptic connectivity by ablating cells within one region and visualizing degenerating axons and terminals using silver nitrate impregnation [15,16,17,18]. While these methods provided foundational insights, they lacked the precision to quantify cell-specific connections. Subsequent advancements introduced retrograde tracers such as Fluoro-Gold, Alexa Fluor 555-conjugated cholera toxin subunit B (CTB), and Fast Blue, which enabled direct labeling of projecting neurons. One such study used CTB and Fast Blue in rats to identify and differentiate two populations of neurons that terminate in the striatum, Corticopontine (CPn) and Crossed Corticostriatal (CCS), with both populations originating within layer V of the cortex [19]. Interestingly, findings show that CCS neurons provide input to CPn neurons via collaterals, suggesting that the CCS neurons also indirectly influence the activity of CPn neurons based on their connectivity [19]. These techniques have significantly enhanced the ability to map corticostriatal pathways. For example, subsequent research using retrograde tracing demonstrated that the prelimbic cortex plays a critical intermediary role in connecting the hippocampus to the dmCPu via polysynaptic pathways [20]. Combined with anterograde tracing methods, such as biotinylated dextran amine, researchers have characterized bidirectional connectivity between cortical and striatal regions with greater accuracy [21]. These important methodological advances have paved the way for more detailed investigations into the topographical organization of prefrontal and adjacent cortical projections to the dmCPu.

Mapping the topographical organization of PFC inputs to the dmCPu requires precision and rigor [22]. Recent studies have utilized advanced analytical models and imaging techniques to quantify these connections, uncovering the roles of specific cortical regions in regulating behavior [23]. Building on earlier findings that established the spatially organized nature of medial and orbital PFC projections to dorsal striatal regions [4,24], our study employed retrograde tracing to map projections specifically to the anterior and posterior dmCPu. By focusing on these critical subdivisions of the dmCPu, we aim to reveal the precise origins of prefrontal and adjacent cortical inputs to regions previously implicated in decision-making and habit formation processes. We utilized Fluoro-Gold, a retrograde tracer known for its high labeling precision and stability, to effectively trace connections from the PFC and relevant adjacent areas to the dmCPu. Additionally, we quantified the percentage of labeled cells across those areas and used heat map visualizations to illustrate patterns of connectivity, providing a comprehensive perspective on critical pathways that support complex behaviors.

## 2. Materials and Methods

### 2.1. Subjects

Eight male Sprague Dawley rats, each weighing between 275–325 g, were obtained from Envigo (Indianapolis, IN, USA). Individual housing was provided for each rat in a temperature-controlled vivarium that maintained a 12 h light/dark cycle, with the light phase starting at 07:00. The rats were given a week-long acclimation period to the vivarium conditions before any experimental procedures began. During acclimation and for the duration of the study, the rats had free access to food and water. All experimental procedures adhered to the ethical guidelines outlined in the Guide for the Care and Use of Laboratory Animals [25] and were approved by the Institutional Animal Care and Use Committee of the University of New Hampshire (protocol #180703; valid from 10/2018 until 10/2021).

### 2.2. Fluoro-Gold Injection Surgery

**Figure 1:**
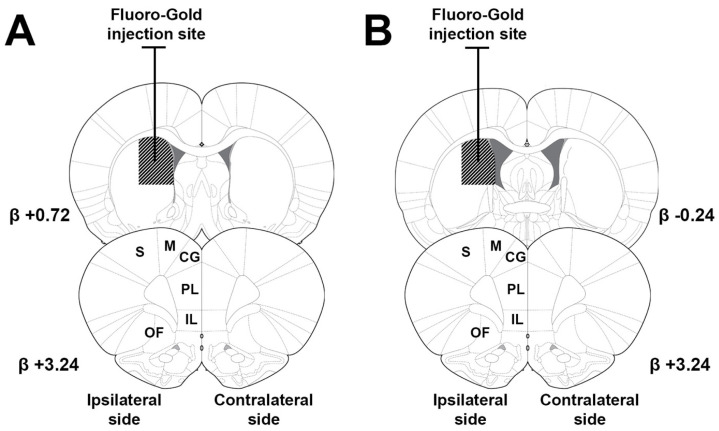
Coronal sections from the rat brain atlas [26] showing targeted areas for Fluoro-Gold injections. (**A**) Anterior dorsomedial caudate–putamen (a-dmCPu) injection site, indicated by diagonal hatching. (**B**) Posterior dorsomedial caudate–putamen (p-dmCPu) injection site, indicated by diagonal hatching. Bottom row illustrates cortical areas (prelimbic cortex [PL], orbitofrontal cortex [OF], motor cortex [M], infralimbic cortex [IL], cingulate gyrus [CG], and somatosensory cortex [S]) from which images were taken for subsequent analyses.

Fluoro-Gold is a retrograde fluorescent tracer utilized to map neural connections. It is absorbed by nerve terminals and transported to the cell bodies, allowing for the identification of neuronal projections. Combined with its fluorescence, this property is critical for delineating neural pathways, enabling clear visualization under microscopy. Figure 1 shows coronal sections from the Paxinos and Watson [26] rat brain atlas. The targeted areas for Fluoro-Gold injections into the anterior dorsomedial caudate–putamen (a-dmCPu; Figure 1A) and posterior dorsomedial caudate–putamen (p-dmCPu; Figure 1B) are shown in the top coronal sections of Figure 1 (see diagonal hatching).

Rats were randomly assigned to receive unilateral Fluoro-Gold injections either in the a- or p-dmCPu (*n* = 4 per group). Anesthesia was induced with 5% isoflurane for 5 min and sustained at 2.5% for the duration of the procedure. Analgesia was provided with butorphanol (5 mg/kg; SC), and meloxicam (0.15 mg/kg; SC) was used for pain management following the induction. Once anesthetized, rats were secured to a stereotaxic frame, and unilateral craniotomies were made under sterile conditions. For a-dmCPu injections, coordinates were set at AP +1.2 mm, ML +2.5 mm, DV −4.3 mm, and for p-dmCPu, at AP −0.35 mm, ML +2.5 mm, DV −4.3 mm [26]. Each subject then received a unilateral injection of 0.2 μL of 2% Fluoro-Gold solution dissolved in 0.9% saline, administered over 10 min. The delivery was controlled by a precision syringe pump (KDS-200; KD Scientific, Holliston, MA, USA), connected to the 28-gauge needle. The needle was maintained in situ for an additional 2 min post-injection to prevent backflow, then slowly retracted over one minute. Post-surgical care included daily observations and administration of butorphanol (5 mg/kg; SC) for additional two consecutive days.

### 2.3. Histological Assessment

Five days after the Fluoro-Gold infusion surgery, rats were euthanized with Euthasol (Virbac, Fort Worth, TX, USA) and perfused with 100 mL 0.02 M sodium phosphate-buffered saline (PBS; 0.9% NaCl, pH 7.4) followed by 300 mL 4% paraformaldehyde solution dissolved in 0.02 M PBS (pH 7.4). Immediately after perfusion, brains were collected and post-fixed in 4% paraformaldehyde for 24 h at 4 °C, followed by cryoprotection in 30% sucrose solution diluted in 0.02 M PBS and stored at 4 °C until saturated. Coronal brain sections of 40 μm of the PFC and CPu were taken from these areas. Injection accuracy in the dorsomedial CPu was confirmed by identifying injection tracks.

For histological analysis, three sections from the PFC (AP +3.2; range +2.8 to +3.6 relative to Bregma) were processed from each rat. Free-floating sections were processed for immunofluorescence following established protocols [27,28,29]. Briefly, sections were washed in 0.02 M PBS (3 × 10 min), blocked with 10% normal goat serum in 0.02 M PBS containing 0.1% Triton X-100 for 1 h at room temperature, and incubated with a 1:5000 dilution of polyclonal rabbit anti-NeuN antibody (ABN78; EMD Millipore; Temecula, CA, USA) for 24 h at 4 °C. After washing with 0.02 M PBS containing 0.1% Triton X-100 (3 × 10 min), sections were incubated with DyLight594 goat anti-rabbit secondary antibody (1:500; Di-1594-1.5; Vector Laboratories, Burlingame, CA, USA) for 2 h at room temperature. This approach allows for simultaneous visualization of the naturally fluorescent retrograde tracer Fluoro-Gold and NeuN-positive neurons (red fluorescence from DyLight594). While Fluoro-Gold typically exhibits yellow-gold fluorescence under UV excitation, it can also be excited by blue light and emit in the green spectrum [30,31], which is how it appears in our figures. This excitation paradigm provides clear differentiation between the green Fluoro-Gold signal and the red NeuN immunofluorescence. The stained sections were washed in 0.02 M PBS, mounted on gelatin-coated slides, and coverslipped with an anti-fade mounting medium containing DAPI (H-1500-10; Vector Laboratories, Burlingame, CA, USA). Finally, all sections were imaged using a Nikon A1R confocal microscope (Nikon, Tokyo, Japan).

**Figure 2:**
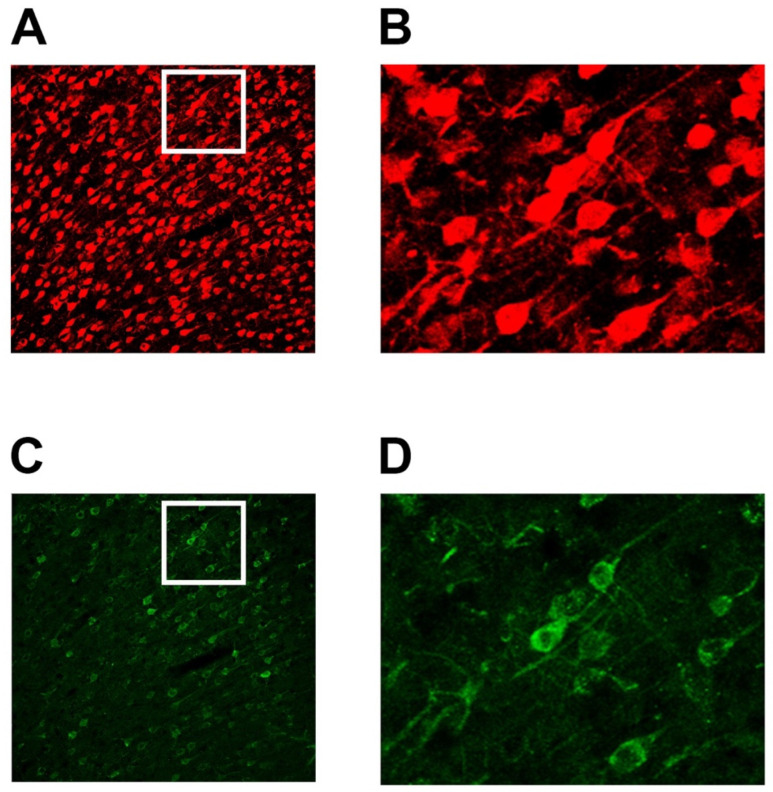
Representative images of cells positively labeled with NeuN and Fluoro-Gold in prefrontal cortex. (**A**) NeuN-positive cells at 20× magnification. (**B**) Magnified view of subsection outlined by a white rectangle in panel (**A**), showing NeuN-positive cells in detail. (**C**) Fluoro-Gold-positive cells at 20× magnification. (**D**) Magnified view of section outlined by a white rectangle in panel (**C**), displaying Fluoro-Gold-positive cells in detail.

Digital images were captured bilaterally from each cortical region of interest (M, S, CG, PL, IL, and OF) across the three coronal sections for each subject. Images were sampled from the epicenter of each area (window size: 636.40 × 636.40 microns; 1024 × 1024 pixels at 20× magnification) as determined by cross-referencing with the rat brain atlas [26]. The entire sampled depth was included in the analysis without focusing on specific cortical layers. This resulted in a total of six images for every brain area per subject: three images from the side ipsilateral to the Fluoro-Gold injection site, and three from the contralateral side. In total, we analyzed 24 coronal sections (3 sections × 8 rats) and 288 images (6 images × 6 cortical regions × 8 rats; 144 images per group). The bottom coronal sections in Figure 1 show the areas from which images were taken for subsequent analyses. Figure 2 shows examples of captured images with cells positively labeled with NeuN and Fluoro-Gold. Panels A and B show NeuN-positive cells, with panel B displaying a magnified view of the subsection outlined by a white rectangle in panel A. Panels C and D depict Fluoro-Gold-positive cells, with panel D providing a magnified view of the section outlined by a white rectangle in panel C. Cells positively labeled with NeuN and Fluoro-Gold-positive cells were quantified using the ‘Object Count’ module within Nikon’s NIS-Elements digital imaging suite. For this process, spectral separation was employed to distinguish Fluoro-Gold from NeuN within 20× magnification z-stacks. These stacks were then transformed into maximum intensity projections, which facilitated automated counting of the labeled cells. This approach enhances the precision and reliability of cell quantification, leveraging the capabilities of this advanced imaging and analysis software.

### 2.4. Analysis

In this study, we employed a comprehensive approach to analyze and present the neural tracing data, focusing on describing the patterns of Fluoro-Gold-positive neurons in the prefrontal cortex and adjacent areas. The cortical areas that were assessed included PL, orbitofrontal cortex (OF), motor cortex (M), IL, cingulate gyrus (CG), and somatosensory cortex (S). We generated descriptive statistics and intuitive heat maps to characterize the distribution of Fluoro-Gold-positive neurons across the factors of Placement, Area, and Side. To supplement these analyses, we employed a mixed-effects model that assessed the main effects and interactions to get a better understanding of how projections from the prefrontal cortex to a- or p-dmCPu differ. By combining detailed descriptive statistics, visually informative heat maps, and a comprehensive mixed-effects model, our data analysis approach offers a thorough and multifaceted examination of the neural tracing data, allowing for a nuanced understanding of the patterns of Fluoro-Gold-positive neurons in the prefrontal cortex and adjacent areas.

Specifically, to evaluate the distribution of neurons projecting to the a- or p-dmCPu, we employed a mixed-effects model. This model analyzed the impact of Side (Ipsilateral vs. Contralateral; within subjects), Fluoro-Gold Placement (Anterior vs. Posterior; within subjects), and Area (between subjects) on the counts of Fluoro-Gold-positive cells. We included Subject as a random effect to address within-subject effects and the repeated nature of the data. The analysis aimed to identify differences in projections to the dmCPu based on Side and Fluoro-Gold Placement, and to explore variations in these projections across different cortical Areas. We also incorporated all two-way and three-way interactions among the fixed effects to understand the complexity of these relationships.

For our statistical analyses, we employed the Python (3.12) framework along with essential libraries for data management, analysis, and visualization. We used the *pandas* (2.2.0) library for data manipulation and aggregation. For statistical analysis, we used the *statsmodels* (0.14.2) library, focusing on its Mixed Linear Model (MixedLM) functionality. This method enabled us to consider both fixed and random effects. Visualization was achieved using *seaborn* (0.13.2) and *matplotlib* (3.9.0) libraries, which were used to generate heat maps. These heat maps visually depict the data’s patterns, specifically illustrating the variations in neuron populations across cortical areas and injection sites based on the average counts of Fluoro-Gold-positive cells.

## 3. Results

**Figure 3:**
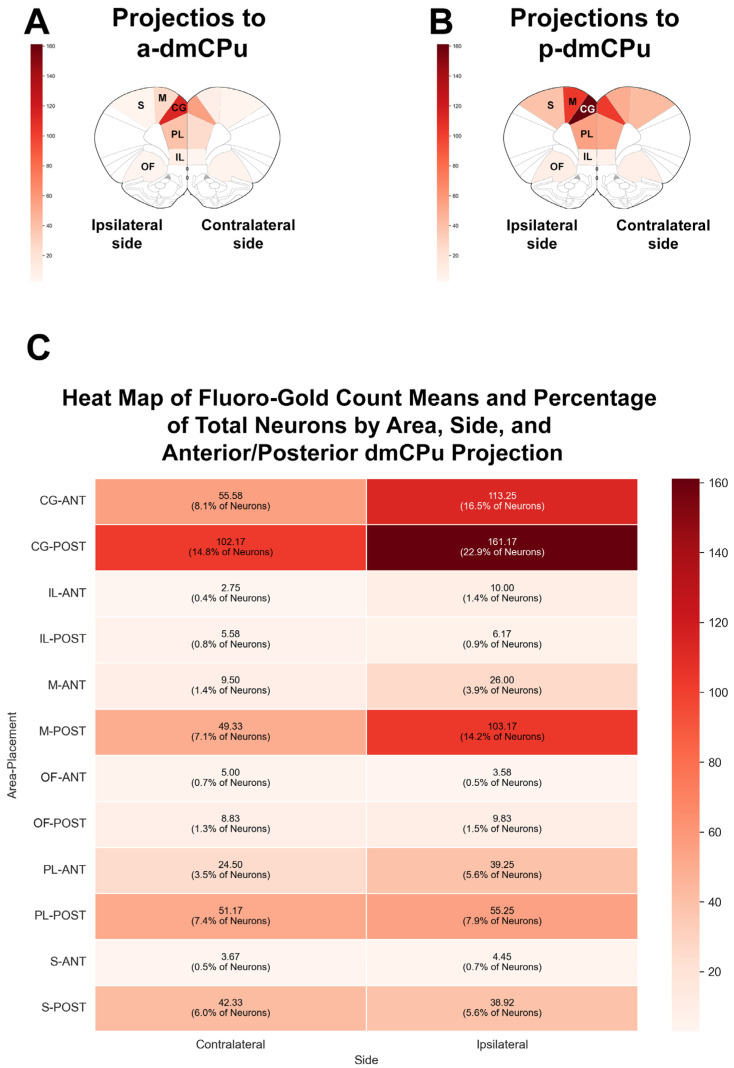
Heat maps illustrating distribution of Fluoro-Gold-positive neurons in examined areas following injections into the anterior or posterior dorsomedial caudate–putamen (dmCPu). (**A**) Heat map of cortical projections to anterior dmCPu (a-dmCPu). (**B**) Heat map of cortical projections to posterior dmCPu (p-dmCPu). (**C**) Combined tabular heat map displaying mean Fluoro-Gold counts and percentage of total neurons in examined areas, organized by Area, Side (ipsilateral or contralateral), and target region of projection (a-dmCPu or p-dmCPu). This tabular format allows for an easy side-by-side visual comparison of the data across different factors. Abbreviations: ANT = anterior, POST = posterior, CG = cingulate cortex, IL = infralimbic cortex, M = motor cortex, OF = orbitofrontal cortex, PL = prelimbic cortex, S = somatosensory cortex.

Histological examination of the injection sites confirmed that all subjects received injections within the predetermined target regions, which are indicated by diagonal hatching in Figure 1. Table 1 presents descriptive statistics from the study, including the means and standard deviations for Fluoro-Gold-positive neurons, total neurons, and the percentage of Fluoro-Gold-positive neurons projecting to either the a- or p-dmCPu. Figure 3 illustrates coronal sections of the prefrontal cortex and adjacent areas with overlaid heat maps for each assessed brain region. Panel A shows the heat map of projections to the a-dmCPu, while panel B displays the heat map of projections to the p-dmCPu. Additionally, panel C of Figure 3 presents a combined heat map in a tabular format, which includes the mean Fluoro-Gold counts and the percentage of total neurons in the sampled regions. This tabular heat map is organized by Area, Side (ipsilateral or contralateral), and the target region of projection (a- or p-dmCPu), facilitating an easy side-by-side visual assessment of the data.

### 3.1. Omnibus Mixed-Effects Analyses

There was a significant main effect of Side, with higher Fluoro-Gold counts on the ipsilateral side compared to the contralateral side (F(1, 276) = 31.58, *p* < 0.001, Cohen’s d = 0.75), suggesting a greater number of projections from the ipsilateral cortex to the dmCPu. The main effect of Placement was also significant, with higher Fluoro-Gold counts in the posterior region of the dmCPu compared to the anterior region (F(1, 276) = 11.83, *p* = 0.001, Cohen’s d = 1.17). The main effect of Area revealed significant differences in Fluoro-Gold counts across brain areas (F(5, 276) = 28.11, *p* < 0.001), with higher counts in CG in comparison to IL, M, OF, PL, and S areas (all *p* < 0.01). A significant two-way interaction between Side and Area (F(5, 276) = 13.88, *p* < 0.001) indicated that the difference in the number of projections between the ipsilateral and contralateral sides varies across cortical areas examined. Other two-way and three-way interactions were not significant (all *p* > 0.05).

In summary, the mixed-effects model revealed that the ipsilateral cortex has a higher number of projections to the dmCPu compared to the contralateral side, and the posterior dmCPu receives more projections than the anterior region. The cingulate cortex showed the highest number of projections to the dmCPu among the cortical areas examined.

### 3.2. Area-Specific Mixed-Effects Analyses

Following the omnibus mixed-effects analyses, we conducted area-specific analyses to further investigate the influence of Side and Placement on Fluoro-Gold-positive cell counts in individual brain areas (Table 2 shows all outputs from those analyses). These analyses provide a more detailed understanding of the patterns of neural projections from each cortical area to the dmCPu.

In the prelimbic cortex, the model revealed a trend towards a main effect of Placement (*p* = 0.158), suggesting that the PL may have a higher number of projections to the posterior dmCPu compared to the anterior region, although this effect did not reach statistical significance. No significant main effect of Side or interaction between Side and Placement was observed in the PL.

The orbitofrontal cortex did not show any significant main effects of Side or Placement, or their interaction, indicating a relatively uniform distribution of projections from the OF to the dmCPu.

In the motor cortex, a significant main effect of Placement (*p* = 0.027) and a significant interaction effect between Side and Placement (*p* = 0.023) were observed. These findings suggest that the motor cortex has a higher number of projections to the posterior dmCPu compared to the anterior region, and that the difference in the number of projections between the ipsilateral and contralateral sides depends on the anterior–posterior location of the injection site.

The infralimbic cortex showed a trend towards a main effect of Side (*p* = 0.068), with the ipsilateral side having a higher number of projections to the dmCPu compared to the contralateral side. No significant main effect of Placement or interaction between Side and Placement was found in the IL.

In the cingulate gyrus, both the main effects of Side (*p* < 0.001) and Placement (*p* = 0.049) were significant, with the ipsilateral side and posterior placement having a substantially higher number of projections to the dmCPu compared to the contralateral side and anterior placement, respectively. No significant interaction between Side and Placement was observed.

Assessment of data from the somatosensory cortex showed a significant main effect of Placement (*p* = 0.044), indicating that the somatosensory cortex has a higher number of projections to the posterior dmCPu compared to the anterior region. No significant main effect of Side or interaction between Side and Placement was detected.

These area-specific analyses reveal diverse patterns of neural projections from the prefrontal and adjacent cortical areas to the dmCPu. The significant findings in the PL, M, CG, and S areas suggest distinct mechanisms underlying the organization of these projections, while the lack of significant effects in the OF and the trend in the IL indicate a more uniform distribution of projections.

## 4. Discussion

This study provides a comprehensive map of prefrontal and adjacent cortical projections to the dorsomedial caudate–putamen, addressing a critical gap in understanding corticostriatal circuit organization. Using retrograde tracing, we identified three key principles: (1) ipsilateral dominance of PFC and adjacent areas projections to the dmCPu, (2) denser cortical input to the posterior compared to the anterior dmCPu, and (3) regional specificity in projections, with the cingulate gyrus (CG) emerging as the most prominent input source. These findings build on earlier anatomical studies by providing detailed, quantitative measures of projection density and regional patterns, e.g., [32,33]. Moreover, our findings align with recent work highlighting corticostriatal circuits’ integrative roles across reward, cognitive, and motor domains [34]. Together, our results offer new insights into the structural foundation of corticostriatal interactions that guide adaptive behavior.

Our analyses revealed distinct patterns in cortical projections to the dmCPu, revealing clear regional and topographical specificity. The cingulate gyrus emerged as the preeminent output area, contributing 22.87% of labeled neurons in ipsilateral projections to the posterior dmCPu, compared to 14.83% contralaterally. This ipsilateral dominance aligns with an established understanding of corticostriatal organization, although it is important to note that the degree of bilateral connectivity in our study varied by region. Projections from the prelimbic cortex accounted for 3.5–7.9% of labeled neurons, whereas the infralimbic cortex exhibited notably sparse inputs (0.4–1.4%). A pronounced anterior–posterior gradient was evident, with motor cortex projections contributing 7.1% to the posterior dmCPu and 1.4% to the anterior region contralaterally, and somatosensory cortex projections following a similar trend (posterior: 6%, anterior: 0.5%). These findings suggest that posterior dmCPu circuits are uniquely suited for integrating sensorimotor information, consistent with their established roles in action selection and behavioral control [6,7,34].

The topographical organization identified in this study builds on and extends previous anatomical research. Our results are consistent with studies demonstrating distinct functional units within the rat corticostriatal system and also provide novel quantitative evidence of previously unrecognized density gradients [2]. The robust cingulate input observed here supports earlier mapping studies of prefrontal projections while adding precise measures of projection density [4]. Similarly, these findings align with detailed analyses of prefrontal projection patterns while offering new quantitative insights [35]. The pronounced anterior–posterior gradient observed in motor and somatosensory projections adds a new dimension to the characterization of the associative striatum [24]. Additionally, the bilateral projections documented in this study, albeit with ipsilateral dominance, complement findings on the importance of bilateral corticostriatal pathways in goal-directed learning [8]. While earlier studies relied on qualitative tracing or functional approaches, this study provides the first systematic quantification of projection densities across multiple prefrontal regions to distinct dmCPu subdomains.

The use of retrograde tracing with Fluoro-Gold in this study provided key advantages for mapping corticostriatal projections, particularly in quantifying projection densities with precision unattainable by earlier methods. Classical degeneration techniques, such as silver impregnation, were instrumental in revealing these pathways [15,16], while anterograde tracers established broader connectivity patterns. However, these approaches lacked the resolution to quantify cell-specific connections. By leveraging the high precision labeling and stability of Fluoro-Gold, combined with a quantitative analytical approach, this study identified not only the presence of projections but also their relative strength across regions. These methods build on earlier retrograde tracing studies that distinguished cortical neuron populations projecting to the striatum, such as Corticopontine and Crossed Corticostriatal neurons [19]. Additionally, while recent polysynaptic circuit mapping techniques using modified rabies viruses offer novel insights [20], our focus on direct projections establishes a critical foundation for understanding the primary architecture of corticostriatal circuits. By combining precise anatomical targeting with systematic quantification, this study represents a methodological advance, bridging the gap between foundational anatomical techniques and modern circuit mapping technologies.

The differential density of cortical projections to anterior and posterior dmCPu substantiates key organizational principles of corticostriatal circuits. The posterior dmCPu received higher input density, particularly from motor and somatosensory areas, highlighting its role in integrating sensorimotor information. This structural organization aligns with previous studies implicating posterior striatal regions in goal-directed actions [6,7]. Recent findings further support corticostriatal gradients as critical for motor and sensory processing, reinforcing the posterior dmCPu’s role in action selection and behavioral regulation [23,34]. The observed bilateral connectivity, though ipsilateral projections dominated, complements evidence that cross-hemispheric coordination supports adaptive behavior [8,9]. The dense cingulate input to both anterior and posterior dmCPu suggests that this region coordinates striatal activity along the anterior–posterior axis. This is consistent with studies linking cingulate–striatal pathways to action–outcome learning and behavioral flexibility [8,34]. Regional specificity in projection patterns may clarify how prefrontal subregions influence striatal processing: prelimbic input may support goal-directed behavior [11], while sparse infralimbic projections may reflect its role in habit formation [13]. Thus, our findings provide a framework for understanding how corticostriatal circuits mediate distinct aspects of behavioral control.

While this study provides detailed insights into corticostriatal circuit organization, several considerations should be noted. The use of male Sprague Dawley rats, though common in anatomical research, limits the generalizability of findings across sex and species. Additionally, while retrograde tracing allows for precise mapping of projection patterns, future studies employing complementary methods, such as anterograde tracers or viral approaches, could further clarify the directional flow and functional dynamics of these circuits. Techniques like optogenetics or chemogenetics, particularly targeting dense cingulate and posterior dmCPu projections, could help establish the causal roles of these pathways in behavior. Such approaches would be especially informative for linking the anterior–posterior gradient observed here to the acquisition and expression of goal-directed actions. Furthermore, examining these circuits in models of psychiatric conditions could reveal circuit-specific pathologies and inform therapeutic strategies. By quantitatively characterizing corticostriatal projections, this study provides a structural framework for understanding how corticostriatal pathways support adaptive behavior and contribute to neuropsychiatric conditions.

## 5. Conclusions

This study provides a comprehensive topographical map of prefrontal cortical and adjacent area projections to the anterior and posterior dorsomedial caudate–putamen in rats. Our findings establish three key organizational principles of corticostriatal circuits: (1) ipsilateral dominance of projections across examined regions, (2) greater projection density to posterior compared to anterior dmCPu, particularly from sensorimotor areas, and (3) regional specificity with cingulate gyrus contributing the densest projections. This anatomical organization supports functional differentiation along the anteroposterior axis of the dmCPu, with anterior regions receiving primarily associative inputs and posterior regions integrating both associative and sensorimotor information. By quantifying projection densities across multiple cortical regions, our approach advances previous qualitative mapping efforts and provides precise anatomical targets for future studies. The detailed projection patterns documented here can guide targeted manipulation of specific corticostriatal pathways using techniques such as dual viral approaches, optogenetics, or chemogenetics. This anatomical foundation offers researchers a roadmap for selectively accessing and manipulating circuits that may be involved in neuropsychiatric conditions associated with corticostriatal dysfunction.

## Figures and Tables

**Table 1:** Descriptive statistics for Fluoro-Gold-positive neurons, total neurons, and percentage of Fluoro-Gold-positive neurons in the prefrontal cortex and adjacent areas.

Placement	Area	Side	Fluoro-Gold-Positive Neurons		Total Neurons		Percentage of Fluoro-Gold-Positive Neurons	
			M	SD	M	SD	M	SD
ANT	CG	CONTRA	55.58	43.93	686.00	23.28	8.10	6.41
		IPSI	113.25	43.72	688.08	32.78	16.46	6.40
	IL	CONTRA	2.75	1.76	752.17	52.20	0.37	0.24
		IPSI	10.00	18.63	733.25	45.01	1.36	2.54
	M	CONTRA	9.50	11.20	691.50	56.40	1.37	1.62
		IPSI	26.00	28.17	674.08	23.63	3.86	4.18
	OF	CONTRA	5.00	1.81	702.92	51.12	0.71	0.26
		IPSI	3.58	1.98	706.92	53.19	0.51	0.28
	PL	CONTRA	24.50	29.33	702.17	32.54	3.49	4.18
		IPSI	39.25	41.65	703.67	38.28	5.58	5.93
	S	CONTRA	3.67	1.23	696.58	34.54	0.53	0.18
		IPSI	4.45	1.29	678.64	19.35	0.66	0.19
POST	CG	CONTRA	102.17	22.48	688.67	37.73	14.84	3.36
		IPSI	161.17	29.51	704.58	34.40	22.87	4.33
	IL	CONTRA	5.58	6.53	728.67	44.47	0.77	0.90
		IPSI	6.17	4.37	719.25	40.51	0.86	0.61
	M	CONTRA	49.33	38.12	692.00	34.97	7.13	5.52
		IPSI	103.17	45.79	729.08	39.46	14.15	6.33
	OF	CONTRA	8.83	6.28	698.75	37.00	1.26	0.90
		IPSI	9.83	11.20	674.00	108.41	1.46	1.68
	PL	CONTRA	51.17	28.40	692.58	27.42	7.39	4.11
		IPSI	55.25	30.56	700.58	23.76	7.89	4.37
	S	CONTRA	42.33	45.90	707.08	43.44	5.99	6.50
		IPSI	38.92	38.24	698.75	30.42	5.57	5.48

**Note:** M = mean, SD = standard deviation, ANT = anterior, POST = posterior, CG = cingulate cortex, IL = infralimbic cortex, M = motor cortex, OF = orbitofrontal cortex, PL = prelimbic cortex, S = somatosensory cortex, CONTRA = contralateral, IPSI = ipsilateral. This table presents mean and standard deviation for Fluoro-Gold-positive neurons, total neurons, and percentage of Fluoro-Gold-positive neurons in sampled areas, organized by Placement, Area, and Side. Data suggest that percentage of Fluoro-Gold-positive neurons is generally higher in the ipsilateral side compared to the contralateral side, and in the posterior placement compared to the anterior placement.

**Table 2:** Mixed-effects model analysis of Fluoro-Gold counts across different brain areas.

Area	Effect	Coefficient	Std. Error	z-Value	*p*-Value	95% CI Lower	95% CI Upper
PL	Side	14.75	10.709	1.377	0.168	−6.239	35.739
PL	Placement	26.667	18.883	1.412	0.158	−10.342	63.676
PL	Side * Placement	−10.667	15.145	−0.704	0.481	−40.35	19.017
OF	Side	−1.417	2.416	−0.586	0.558	−6.152	3.319
OF	Placement	3.833	3.274	1.171	0.242	−2.583	10.249
OF	Side * Placement	2.417	3.417	0.707	0.479	−4.28	9.113
M	Side	16.5	11.584	1.424	0.154	−6.205	39.205
M	Placement	39.833	18.025	2.21	**0.027**	4.505	75.162
M	Side * Placement	37.333	16.383	2.279	**0.023**	5.223	69.443
IL	Side	7.25	3.968	1.827	*0.068*	−0.527	15.027
IL	Placement	2.833	4.669	0.607	0.544	−6.317	11.984
IL	Side * Placement	−6.667	5.612	−1.188	0.235	−17.665	4.332
CG	Side	57.667	9.455	6.099	**0.000**	39.135	76.198
CG	Placement	46.583	23.637	1.971	**0.049**	0.255	92.912
CG	Side * Placement	1.333	13.371	0.1	0.921	−24.874	27.541
S	Side	0.743	8.545	0.087	0.931	−16.005	17.491
S	Placement	38.667	19.162	2.018	**0.044**	1.11	76.223
S	Side * Placement	−4.16	11.935	−0.349	0.727	−27.551	19.232

**Note:** PL denotes prelimbic cortex, OF represents orbitofrontal cortex, M stands for motor cortex, IL refers to infralimbic cortex, CG indicates cingulate gyrus, and S signifies somatosensory cortex. * denotes interaction. *p*-values in bold are less than 0.05, indicating statistical significance. *p*-values in italic are less than 0.1, suggesting a trend towards significance.

## Data Availability

The data presented in this study are available on request from the corresponding author due to ethical and institutional guidelines regarding the handling of animal research data.

## References

[1] Friedman N.P., Robbins T.W. (2022). The Role of Prefrontal Cortex in Cognitive Control and Executive Function. Neuropsychopharmacology.

[2] Mailly P., Aliane V., Groenewegen H.J., Haber S.N., Deniau J.-M. (2013). The Rat Prefrontostriatal System Analyzed in 3D: Evidence for Multiple Interacting Functional Units. J. Neurosci..

[3] Sesack S.R., Deutch A.Y., Roth R.H., Bunney B.S. (1989). Topographical Organization of the Efferent Projections of the Medial Prefrontal Cortex in the Rat: An Anterograde Tract-tracing Study with *Phaseolus vulgaris* Leucoagglutinin. J. Comp. Neurol..

[4] Berendse H.W., Graaf Y.G., Groenewegen H.J. (1992). Topographical Organization and Relationship with Ventral Striatal Compartments of Prefrontal Corticostriatal Projections in the Rat. J. Comp. Neurol..

[5] Hunnicutt B.J., Jongbloets B.C., Birdsong W.T., Gertz K.J., Zhong H., Mao T. (2016). A Comprehensive Excitatory Input Map of the Striatum Reveals Novel Functional Organization. eLife.

[6] Yin H.H., Ostlund S.B., Balleine B.W. (2008). Reward-Guided Learning beyond Dopamine in the Nucleus Accumbens: The Integrative Functions of Cortico-Basal Ganglia Networks. Eur. J. Neurosci..

[7] Yin H.H., Ostlund S.B., Knowlton B.J., Balleine B.W. (2005). The Role of the Dorsomedial Striatum in Instrumental Conditioning. Eur. J. Neurosci..

[8] Hart G., Bradfield L.A., Fok S.Y., Chieng B., Balleine B.W., Hart G., Bradfield L.A., Fok S.Y., Chieng B., Balleine B.W. (2018). The Bilateral Prefronto-Striatal Pathway Is Necessary for Learning New Goal-Directed Actions. Curr. Biol..

[9] Peak J., Hart G., Balleine B.W. (2019). From Learning to Action: The Integration of Dorsal Striatal Input and Output Pathways in Instrumental Conditioning. Eur. J. Neurosci..

[10] Bradfield L.A., Hart G., Balleine B.W. (2018). Inferring Action-Dependent Outcome Representations Depends on Anterior but Not Posterior Medial Orbitofrontal Cortex. Neurobiol. Learn. Mem.

[11] Corbit L.H., Balleine B.W. (2003). The Role of Prelimbic Cortex in Instrumental Conditioning. Behav. Brain Res..

[12] Ostlund S.B., Balleine B.W. (2005). Lesions of Medial Prefrontal Cortex Disrupt the Acquisition but Not the Expression of Goal-Directed Learning. J. Neurosci..

[13] Coutureau E., Killcross S. (2003). Inactivation of the Infralimbic Prefrontal Cortex Reinstates Goal-Directed Responding in Overtrained Rats. Behav. Brain Res..

[14] Killcross S., Coutureau E. (2003). Coordination of Actions and Habits in the Medial Prefrontal Cortex of Rats. Cereb. Cortex.

[15] Fink R.P., Heimer L. (1967). Two Methods for Selective Silver Impregnation of Degenerating Axons and Their Synaptic Endings in the Central Nervous System. Brain Res..

[16] Frotscher M., Rinne U., Hassler R., Wagner A. (1981). Termination of Cortical Afferents on Identified Neurons in the Caudate Nucleus of the Cat: A Combined Golgi-EM Degeneration Study. Exp. Brain Res..

[17] Hoff E.C., Sherrington C.S. (1997). Central Nerve Terminals in the Mamalian Spinal Cord and Their Examination by Experimental Degeneration. Proc. R. Soc. Lond. Ser. B Contain. Pap. Biol. Character.

[18] Nauta W.J.H., Gygax P.A. (1951). Silver Impregnation of Degenerating Axon Terminals in the Central Nervous System: (1) Technic. (2) Chemical Notes. Stain Technol..

[19] Morishima M., Kawaguchi Y. (2006). Recurrent Connection Patterns of Corticostriatal Pyramidal Cells in Frontal Cortex. J. Neurosci..

[20] Du W., Li E., Guo J., Arano R., Kim Y., Chen Y.-T., Thompson A., Oh S.J., Samuel A., Li Y. (2023). Directed Stepwise Tracing of Polysynaptic Neuronal Circuits with Replication-Deficient Pseudorabies Virus. Cell Rep. Methods.

[21] Parent M., Parent A. (2006). Single-axon Tracing Study of Corticostriatal Projections Arising from Primary Motor Cortex in Primates. J. Comp. Neurol..

[22] Yao F., Zhang E., Gao Z., Ji H., Marmouri M., Xia X. (2018). Did You Choose Appropriate Tracer for Retrograde Tracing of Retinal Ganglion Cells? The Differences between Cholera Toxin Subunit B and Fluorogold. PLoS ONE.

[23] Peak J., Chieng B., Hart G., Balleine B.W. (2020). Striatal Direct and Indirect Pathway Neurons Differentially Control the Encoding and Updating of Goal-Directed Learning. eLife.

[24] Reep R.L., Cheatwood J.L., Corwin J.V. (2003). The Associative Striatum: Organization of Cortical Projections to the Dorsocentral Striatum in Rats. J. Comp. Neurol..

[25] (2011). Guide for the Care and Use of Laboratory Animals.

[26] Paxinos G., Watson C. (2007). The Rat Brain in Stereotaxic Coordinates.

[27] Charntikov S., Pittenger S.T., Swalve N., Li M., Bevins R.A. (2017). Double Dissociation of the Anterior and Posterior Dorsomedial Caudate-Putamen in the Acquisition and Expression of Associative Learning with the Nicotine Stimulus. Neuropharmacology.

[28] Kim K.K., Adelstein R.S., Kawamoto S. (2009). Identification of Neuronal Nuclei (NeuN) as Fox-3, a New Member of the Fox-1 Gene Family of Splicing Factors. J. Biol. Chem..

[29] Mullen R.J., Buck C.R., Smith A.M. (1992). NeuN, a Neuronal Specific Nuclear Protein in Vertebrates. Development.

[30] Schmued L.C., Fallon J.H. (1986). Fluoro-Gold: A New Fluorescent Retrograde Axonal Tracer with Numerous Unique Properties. Brain Res..

[31] Wessendorf M.W. (1991). Fluoro-Gold: Composition, and Mechanism of Uptake. Brain Res..

[32] Corbit L.H., Janak P.H. (2007). Inactivation of the Lateral but Not Medial Dorsal Striatum Eliminates the Excitatory Impact of Pavlovian Stimuli on Instrumental Responding. J. Neurosci..

[33] Yin H.H., Knowlton B.J., Balleine B.W. (2004). Lesions of Dorsolateral Striatum Preserve Outcome Expectancy but Disrupt Habit Formation in Instrumental Learning. Eur. J. Neurosci..

[34] Haber S.N. (2016). Corticostriatal Circuitry. Dialogues Clin. Neurosci..

[35] Gabbott P.L.A.A., Warner T.A., Jays P.R.L.L., Salway P., Busby S.J. (2005). Prefrontal Cortex in the Rat: Projections to Subcortical Autonomic, Motor, and Limbic Centers. J. Comp. Neurol..

